# Sedentary behaviour in relation to ovarian cancer risk: a systematic review and meta-analysis

**DOI:** 10.1007/s10654-020-00712-6

**Published:** 2021-01-25

**Authors:** Veronika S. Biller, Michael F. Leitzmann, Anja M. Sedlmeier, Felix F. Berger, Olaf Ortmann, Carmen Jochem

**Affiliations:** 1 Department of Epidemiology and Preventive Medicine, University of Regensburg, Regensburg, Germany; 2 Department of Gynaecology and Obstetrics, University of Regensburg, Regensburg, Germany

**Keywords:** Sedentary behaviour, Ovarian cancer, Meta-analysis, Systematic review

## Abstract

Sedentary behaviour is an emerging risk factor for several site-specific cancers. Ovarian cancers are often detected at late disease stages and the role of sedentary behaviour as a modifiable risk factor potentially contributing to ovarian cancer risk has not been extensively examined. We systematically searched relevant databases from inception to February 2020 for eligible publications dealing with sedentary behaviour in relation to ovarian cancer risk. We conducted a systematic review and meta-analysis, calculating summary relative risks (RR) and 95% confidence intervals (CI) using a random-effects model. We calculated the E-Value, a sensitivity analysis for unmeasured confounding. We tested for publication bias and heterogeneity. Seven studies (three prospective cohort studies and four case–control studies) including 2060 ovarian cancer cases were analysed. Comparing highest versus lowest levels of sedentary behaviour, the data indicated a statistically significant increase in the risk of ovarian cancer in relation to prolonged sitting time (RR = 1.29, 95% CI = 1.07–1.57). Sub-analyses of prospective cohort studies (RR = 1.33, 95% CI = 0.92–1.93) and case–control studies (RR = 1.28, 95% CI = 0.98–1.68) showed statistically non-significant results. Sensitivity analysis showed that an unmeasured confounder would need to be related to sedentary behaviour and ovarian cancer with a RR of 1.90 to fully explain away the observed RR of 1.29. Our analyses showed a statistically significant positive association between sedentary behaviour and ovarian cancer risk.

**Supplementary Information:**

The online version of this article (10.1007/s10654-020-00712-6).

## Introduction

Ovarian cancer is the eighth most common cancer in women worldwide, with 295,414 incident cases and 184,799 deaths in 2018 [1]. Due to non-specific symptoms and a lack of effective screening methods, ovarian cancer is often detected at an advanced stage, which results in poor survival rates [2, 3].

Sedentary behaviour is a modifiable risk factor for several chronic diseases including different tumour entities [4]. Sedentary behaviour is defined as “any waking behaviour characterized by an energy expenditure ≤ 1.5 metabolic equivalents (METs), while in a sitting, reclining or lying posture” [5]. Individuals spend more than half of their daily waking hours sedentary, e.g. while watching television (TV), using means of transport, at work or in school [6]. Importantly, sedentary behaviour is distinct from physical inactivity since someone can meet the physical activity recommendations despite being highly sedentary throughout the remaining waking hours [7].

Several epidemiologic studies have investigated the association between sedentary behaviour and ovarian cancer risk. Previous meta-analyses generated inconsistent findings [4, 8–10]. Whereas two initial meta-analyses published in 2014 yielded positive but statistically non-significant results [4, 10], two recent meta-analyses showed a statistically significant positive association between prolonged sitting time and ovarian cancer risk [8, 9]. It is noteworthy that none of the meta-analyses included all available studies with ovarian cancer as the primary outcome and the majority assessed multiple cancer types [4, 9, 10]. The most recent meta-analysis did not exclusively investigate sedentary behaviour as exposure and entailed several methodological limitations [8]. Therefore, an up-to-date and comprehensive systematic review and meta-analysis focusing on sedentary behaviour in relation to ovarian cancer risk is needed.

## Methods

The present study was performed according to the Preferred Reporting Items for Systematic Reviews and Meta-Analyses (PRISMA) [11]. The PRISMA checklist can be accessed online (Online Resource 1).

### Inclusion criteria

To qualify for our analysis, studies had to fulfil the following inclusion criteria: studies 1) represented a cohort or case–control design, 2) were conducted in adult women, 3) defined total sitting time, leisure-time sitting, TV-viewing time or occupational sitting time as an exposure variable and considered ovarian cancer risk as the primary outcome, 4) provided a relative risk (RR), odds ratio (OR) or hazard ratio (HR) and 95% confidence intervals (CI) for highest versus lowest levels of sedentary behaviour and 5) were published in English language.

We excluded studies assessing physical inactivity, as physical inactivity cannot be equated with sedentary behaviour [7]. Furthermore, studies that considered ovarian cancer mortality or survival instead of ovarian cancer risk as the primary outcome were also unsuitable for analysis. We did not consider editorials, comments, letters, guidelines or news articles. All inclusion and exclusion criteria were defined prior to conducting the review.

### Search strategy

We systematically searched the PubMed and Web of Science databases from inception to February 2020. Weekly updates provided by both databases were used and we reiterated our search on a monthly basis. Additionally, we manually reviewed the reference lists of suitable articles and consulted with experts in the field to obtain further relevant publications. Two authors (VB and CJ) developed the search term (Online Resource 2), which entailed sedentary behaviour and appropriate synonyms, as well as terms related to sedentary behaviours (e.g. leisure-time sitting, TV-viewing, computer use, transport time or reading), combined with keywords for ovarian neoplasms or site-specific cancer.

The first author (VB) screened titles and abstracts and retrieved full-text articles that met the inclusion criteria for further reading. The final decision about inclusion in the meta-analysis was made by two authors (VB and CJ) and disagreements were resolved by discussion with ML. If several reports regarding the same cohort study were found, we included the most recent publication.

### Data extraction

The following data of each study was extracted by the first author (VB) and re-examined by the second author (CJ): first author’s name, publication year, study design and name, size and age of study population, geographic region, follow-up time, number of incident cases, number of controls (if case–control design), histologic cancer type (total, epithelial, serous, non-serous), case ascertainment (self-report, medical record, linkage with state registries), sedentary behaviour domain (total, leisure-time, TV-viewing time, occupational time (hours per day spent sitting)), exposure ascertainment (self-administered questionnaire, standardised interview, job title assignment), unit of measurement (frequency, duration, intensity), and adjustment factors, risk estimates (RR, OR, HR) with corresponding 95% CIs.

### Statistical methods

Risk estimates were interpreted as relative risks (RR_i_). We computed the natural logarithms of relative risks (log(RR_i_)) with their corresponding standard errors (s_i_ = d_i_ / 1.96), where d_i_ was defined as maximum of (log(upper bound 95% CI of RR_i_) – log(RR_i_)) and (log(RR_i_) – (log(lower bound 95% CI of RR_i_)). The logarithmic relative risks were weighted by ω_i_ = 1/(s_i_^2^ + τ_i_^2^) using a random-effects model, where s_i_ describes the standard error of log(RR_i_) and τ_I_ the restricted maximum likelihood estimate of the overall variance allowing for effect measure heterogeneity [12].

In the primary meta-analysis, we included one risk estimate per study. We preferred risk estimates primarily assessing total sitting time [13–15] or leisure-time sitting [16, 17] as exposure variables. We included two risk estimates of studies assessing solely TV-viewing time [18] or occupational sitting time [19], respectively. For all analyses, we chose the maximally adjusted risk estimate. We tested for heterogeneity using the Q- and I^2^-statistics [20].

We performed a priori defined stratified analyses with meta-regression random-effects meta-analysis and investigated the influence of study design (prospective cohort studies, case–control studies), geographic region (Asia, Europe, North America), sedentary behaviour domain (total, leisure-time, TV-viewing time, occupational time), exposure ascertainment (self-administered questionnaire, standardised interview, job title assignment) and adjustments for body mass index (BMI), family history of breast or ovarian cancer, parity, age at menarche, age at menopause, use of oral contraceptives, hormone therapy, education, alcohol use and smoking status.

Publication bias was evaluated using a funnel plot [21], Begg’s rank correlation test [22] and Egger’s regression test [21]. Additionally, we performed outlier and influence diagnostics and leave-one-out analysis [23].

We calculated the E-Value to estimate how strong an unmeasured confounder would need to be to fully resolve a reported exposure-outcome association, above and beyond measured covariates [24]. As part of the calculation, we quantified the size of unobserved confounding able to nullify the mean risk ratio. As a meta-analytic extension of the E-Value, we calculated the unmeasured confounding strengths sufficient to allow 10% to 50% of studies with true RR above a meaningful scientific threshold (i.e., RR > 1.10) to remain statistically significant [25].

All risk estimates were calculated with the corresponding 95% CIs. P-values < 0.05 were considered statistically significant. All statistical analyses were performed with the software R (version 3.5.1) [26], using the packages “robumeta” [27], “metafor” [12], “EValue” [25] and “MetaUtility” [28].

## Results

### Study selection

**Fig. 1:**
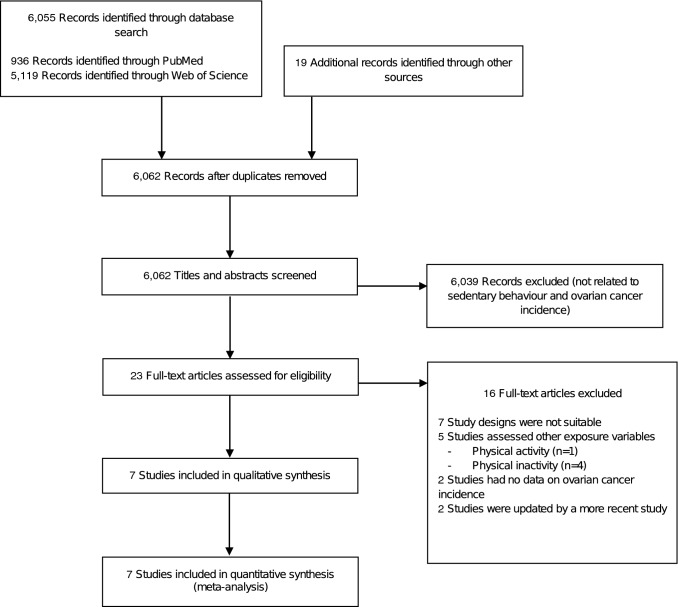
PRISMA flow diagram depicting the process of study selection for meta-analysis

The systematic literature search identified 6074 publications, of which 6055 were found through electronic literature search (936 records through PubMed, 5119 records through Web of Science) and 19 through hand search or other sources (Fig. 1). After removal of duplicates, we screened titles and abstracts of 6062 publications. We assessed 23 full-text articles for eligibility. Of these, seven studies used incompatible study designs (meta-analyses or systematic reviews) [4, 8–10, 29–31], five studies investigated other exposure variables (physical activity or physical inactivity) [32–36], two studies did not provide sufficient data on ovarian cancer risk [37, 38] and two studies were updated by a more recent publication [39, 40] (Online Resource 3). After exclusion of these studies, a total of seven eligible articles, containing three prospective cohort studies [14, 16, 18] and four case–control studies [13, 15, 17, 19], were included in our systematic review and meta-analysis.

### Study characteristics

The main characteristics of the seven studies included are presented in Table 1. In total, the studies comprised 197,416 participants and 2060 ovarian cancer cases. Three studies provided risk estimates for total sitting time [13–15], three for leisure-time sitting [15–17] or TV-viewing time [14, 15, 18], respectively, and two studies for occupational sitting time [15, 19]. Two studies originated from Europe [17, 19], three from Asia [13, 15, 18] and two from the United States [14, 16]. Three studies assessed sedentary behaviour through a self-administered questionnaire [14, 16, 18], three studies used a standardised interview [13, 15, 17], while one study used job titles [19]. The number of adjustment factors varied between two and 17 variables per study. All seven studies used daily duration of sedentary behaviour as exposure variable. The ovarian cancer cases were identified by self-report [16], medical records [13, 15–19], linkage to state cancer registries [14, 16, 18] or death certificate [16]. We preferred risk ratios for total ovarian cancer [16, 18, 19] or, if not provided, epithelial ovarian cancer [13–15, 17].

**Table 1:** Study characteristics of the three cohort studies and four case–control studies of sedentary behaviour and ovarian cancer risk

First author, year, country	Sample population and follow-up time	Number of cases and method of assessment	Assessment and categorisation of sedentary behaviour	Exposure, ovarian cancer endpoint and risk estimate included in main analysis	Confounding variables
Prospective cohort studies					
Ukawa et al., 2018, Japan [18]	34 758 women from the JACC Study, aged 40–79 years at baseline. Mean follow-up 19.4 years	59 incident ovarian cancer cases identified by local medical records and linkage to state cancer registries	Self-reported TV-viewing time, categorised as < 2, 2–2.9, 3–3.9, 4–4.9 or ≥ 5 h/day	TV-viewing time ≥ 5 h/day, total OCa: HR = 1.81 [0.75–4.39]	Age, college education, family history of breast, ovarian or prostate cancer, body mass index, daily walking time, smoking status, alcohol consumption, parity (number of live births), age at menopause, age at menarche, hormone therapy, daily sleeping time
Hildebrand et al., 2015, USA [16]	63 972 women from the ACS CPSII-Nutrition Cohort, aged 50–74 years at baseline. Mean follow-up 19 years	651 incident ovarian cancer cases identified by self-report and verified by medical records or linkage with state cancer registries, or by death certificate, and verified through registries	Self-reported leisure-time sitting, categorised as < 3, 3–5 or ≥ 6 h/day. Histological differentiation in total, serous and non-serous ovarian cancer	Leisure-time sitting ≥ 6 h/day, total OCa: RR = 1.44 [1.12–1.85]	Age, education, body mass index, smoking status, parity (number of live births), use of oral contraceptives, postmenopausal hormone use
update of: Patel et al., 2015, USA [39]					
Patel et al., 2006, USA [40]					
Xiao et al., 2013, USA [14]	96 247 women from the NIH-AARP Diet and Health Study aged 50–71 years at baseline. Mean follow-up 11 years	467 incident ovarian cancers identified by linkage with 10 state cancer registries	Self-reported total sitting time, categorised as < 3, 3–4, 5–6 or ≥ 7 h/day	Total sitting time ≥ 7 h/day, epithelial OCa:	Age, race, education, smoking, parity (number of live births), age at menopause, age at menarche, oral contraceptive use, postmenopausal hormone use, marital status
			Self-reported TV-viewing time, categorised as < 3, 3–4, 5–6 or ≥ 7 h/day	RR = 1.06 [0.81–1.39]	
Case–control studies					
Gazibara et al., 2013, Serbia [17]	80 ovarian cancer cases treated at the national referral centres for ovarian cancer Belgrade, Serbia, (mean age 56.1 years) and 160 cancer controls (mean age 56.7 years) between 2006–2008	80 ovarian cancer cases	Standardised interview, leisure-time sitting, categorised as ≤ 3 or ≥ 4 h/day	Leisure-time sitting ≥ 4 h/day, epithelial OCa:	Age, municipality of residence (matching factors)
				OR = 1.8 [1.0–3.1]	
Lee et al., 2013, China [13]	500 ovarian cancer cases treated in four public hospitals in Guangzhou, China, (mean age 59.07 years) and 500 cancer controls (mean age 59.71 years) between 2006–2008	500 ovarian cancer cases	Standardised interview, total sitting time, categorised as ≤ 4, 4,5–8 or ≥ 8.5 h/day	Total sitting time ≥ 8,5 h/day, epithelial OCa:	Age, education level, family history of ovarian or breast cancer, body mass index, smoking status, parity, menopausal status, oral contraceptive use;
				OR = 1.07 [0.77–1.48]	
Zhang et al., 2004, China [15]	254 ovarian cancer cases diagnosed in the past 3 years in Zhejiang province, China, (mean age 46.8 years) and 652 cancer controls (mean age 48 years)	254 ovarian cancer cases	Standardised interview, occupational sitting time, categorised as < 2, 2–6 and > 6 h/day	Total sitting time > 10 h/day, epithelial OCa:	Age, education, locality, family history of ovarian cancer, body mass index, physical activity, smoking status, alcohol consumption, tea drinking, total energy intake, parity, menopausal status, oral contraceptive use, hormone therapy, tubal ligation, marital status, family income;
			Standardised interview, TV-viewing time, categorised as < 2, 2–4 or > 4 h/day		
			Standardised interview, other sedentary activities, categorised < 2, 2–4 or > 4 h/day	OR = 1.77 [1.0–3.1]	
			Standardised interview, total sitting time, categorised as < 4, 4–10 or > 10 h/day		
Dosemeci et al., 1993, Turkey [19]	49 ovarian cancer cases treated at an oncological treatment centre in Istanbul, Turkey, and 244 cancer controls	49 ovarian cancer cases	Job titles, occupational sitting time, categorised as < 2, 2–6 and > 6 h/day	Occupational sitting time > 6 h/day, total OCa:	Age, smoking, socioeconomic status
				OR = 0.4 [0.1–1.9]	

*OCa* ovarian cancer, *JACC Study* Japan Collaborative Cohort Study for Evaluation of Cancer Risk, *ACS CPS-II Nutrition Cohort* American Cancer Society Cancer Prevention Study II Nutrition Cohort, *NIH-AARP Diet and Health Study* National Institute of Health-American Association of Retired Persons Diet and Health Study, *TV* television, *BMI* body mass index, *hours/day* hours per day, *kg/m2* kilogram per square meter, *HR* hazard ratio, *RR* relative risk, *OR* odds ratio, values in brackets indicate the corresponding 95% confidence interval (CI)

### Sedentary behaviour and ovarian cancer risk

**Fig. 2:**
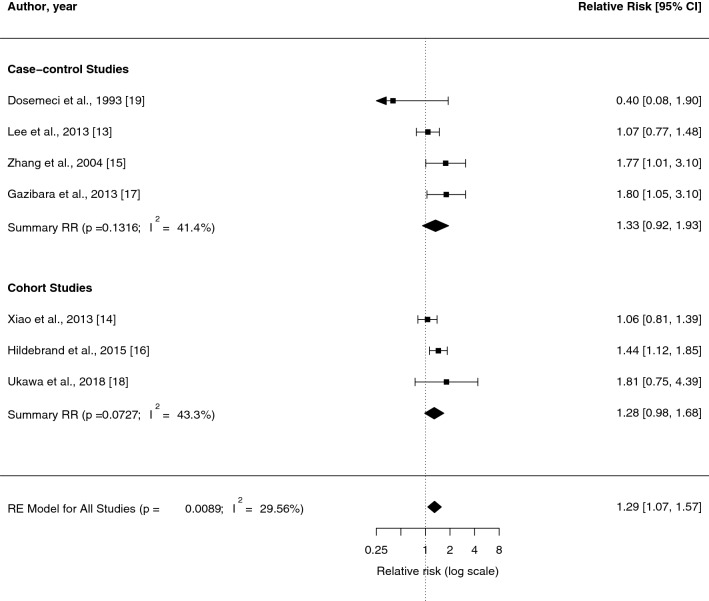
Forest plot of random-effects meta-analysis of adjusted risk estimates of high versus low sedentary behaviour in relation to ovarian cancer risk. The black square and the respective line represent the risk estimate and corresponding 95% confidence interval (CI) for each study. The diamond represents the summary relative risk with the corresponding CI for ovarian cancer risk based on case–control studies, cohort studies, and all studies combined, respectively. P, P-value (statistical significance); I^2^, heterogeneity among studies; RR, relative risk; RE Model, random effects mode

Our primary random-effects meta-analysis of seven risk estimates revealed a statistically significant positive association between high versus low level of sedentary behaviour and risk of ovarian cancer (RR = 1.29, 95% CI = 1.07–1.57) (Fig. 2). There was low heterogeneity among these studies (I^2^ = 29.56%, P-heterogeneity = 0.1523). The summary risk estimates were almost identical in cohort (RR = 1.33, 95% CI = 0.92–1.93) and case–control (RR = 1.28, 95% CI = 0.98–1.68) studies, although the stratum-specific risk estimates were statistically non-significant and heterogeneity was modest in both cohort (I^2^ = 41,4%, P-heterogeneity = 0.1316) and case–control (I^2^ = 43,3%, P-heterogeneity = 0.0727) studies.

### Stratified analyses and sensitivity analyses

Stratified sub-analyses showed that the relation of sedentary behaviour to ovarian cancer risk was not modified by study design, geographic region, sedentary behaviour domain, exposure ascertainment, or various adjustment factors, including adjustments for BMI, family history of breast or ovarian cancer, parity, age at menarche, age at menopause, oral contraceptives, hormone therapy, education, alcohol use and smoking status (all P-difference > 0.05) (Table 2).

**Table 2:** Stratification criteria, relative risk, difference between included ovarian cancer risk studies and results of random-effects meta-regression meta-analysis for each subgroup

Stratification criteria	Number of included RRs	RR (high versus low SB)	95% CI	I^2^ (%)	P(diff)
Total incident ovarian cancer	7	1.29	1.07, 1.57	29.56	NA
*Study design*					
Prospective cohort studies	3	1.28	0.98, 1.68	43.26	
Case–control studies	4	1.33	0.92, 1.93	41.42	0.9021
*Geographic region*					
Asia	3	1.36	0.91, 2.03	39.80	
Europe	2	1.02	0.24, 4.26	68.66	
North America	2	1.24	0.92, 1.68	62.22	0.9104
*Sedentary behaviour domain*					
Total	3	1.13	0.93, 1.38	0.01	
Leisure-time	3	1.34	0.95, 1.90	45.02	
TV-viewing time	3	1.54	0.79, 2.97	51.78	
Occupational time	2	1.06	0.23, 4.83	72.50	0.9018
*Exposure ascertainment*					
Self-administered questionnaire	3	1.28	0.98, 1.68	43.26	
Standardised interview	3	1.42	0.97, 2.07	49.31	
Job title assignment	1	0.40	0.08, 1.90	NA	0.3232
*Adjustment for BMI*					
Adjusted for BMI	4	1.36	1.08, 1.71	23.59	
Not adjusted for BMI	3	1.18	0.71, 1.95	54.24	0.5031
*Adjustment for family history of breast or ovarian cancer*					
Adjusted for family history	3	1.36	0.91, 2.03	39.80	
Not adjusted for family history	4	1.28	0.98, 1.68	43.24	0.8421
*Adjustment for parity*					
Adjusted for parity	5	1.27	1.04, 1.54	31.24	
Not adjusted for parity	2	1.02	0.24, 4.26	68.66	0.6129
*Adjustment for age at menarche / menopause*					
Adjusted for age at menarche / menopause	2	1.16	0.78, 1.74	21.94	
Not adjusted for age at menarche / menopause	5	1.36	1.09, 1.70	23.85	0.4008
*Adjustment for oral contraceptives*					
Adjusted for oral contraceptives	4	1.25	1.02, 1.53	37.31	
Not adjusted for oral contraceptives	3	1.60	1.02, 2.49	0.00	0.3749
Adjustment for hormone therapy					
Adjusted for hormone therapy	4	1.35	1.05, 1.73	39.51	
Not adjusted for hormone therapy	3	1.19	0.72, 1.97	51.36	0.6191
*Adjustment for education*					
Adjusted for education	5	1.27	1.04, 1.54	31.24	
Not adjusted for education	2	1.02	0.24, 4.26	68.66	0.6129
*Adjustment for alcohol use*					
Adjusted for alcohol use	2	1.78	1.11, 2.86	0.00	
Not adjusted for alcohol use	5	1.23	1.00, 1.51	33.90	0.1777
*Adjustment for smoking status*					
Adjusted for smoking status	6	1.24	1.02, 1.51	26.55	
Not adjusted for smoking status	1	1.80	1.05, 3.10	NA	0.2468

*RR* relative risk, *SB* sedentary behaviour, *CI* confidence interval, *I*^2^ heterogeneity among studies, *P(diff)* P value for difference in the result of moderator analysis, *NA* not applicable, *BMI* body mass index

Visual inspection of the funnel plot yielded no evidence for publication bias (Online Resource 4), as indicated by Egger’s regression test (P = 0.97) and Begg’s correlation test (P = 1.00).

Leave-one-out diagnostics and influence diagnostics of the seven included studies showed no relevant changes in summary risk estimates, spanning a range from RR = 1.24 (95% CI = 1.02–1.51) to RR = 1.38 (95% CI = 1.12–1.70). For example, when the case–control study that used job title assignment and occupational sitting time as exposure variable [17] was omitted from the analysis, results showed a summary risk estimate of RR = 1.32 (95% CI = 1.08–1.60). When we excluded the prospective cohort study that assessed only TV-viewing time as exposure variable [18], the summary risk estimate was RR = 1.27 (95% CI = 1.04–1.55).

The sensitivity analysis for unmeasured confounding showed that an unobserved confounder needed to be associated with both sedentary behaviour and ovarian cancer with a risk ratio of at least 1.90 to fully explain away the mean RR of 1.29, above and beyond the measured confounders, yet weaker confounding could not do so. To render the estimated risk ratio statistically non-significant, unobserved confounding strength associated with sedentary behaviour and ovarian cancer risk with a risk ratio of 1.34 would be necessary to move the lower confidence limit of 1.07 to include the null. Unobserved confounder strengths with RRs of 2.14, 1.96, 1.84, 1.73 and 1.63 in each meta-analysed study, respectively, would be necessary to reduce to less than 10%, 20%, 30%, 40%, and 50%, respectively, the percentage of studies with true causal RRs above the meaningful scientific threshold of 1.10 (Online Resource 5).

## Discussion

The results of our primary meta-analysis, including 2060 ovarian cancer cases from seven studies, showed a 29% increase in ovarian cancer risk with high versus low levels of sedentary behaviour. The association between sedentary behaviour and ovarian cancer risk was not modified by study design, geographic region, sedentary behaviour domain, exposure ascertainment, or adjustment for several confounders, including reproductive, hormonal, and lifestyle factors.

Our results update and expand the existing evidence on sedentary behaviour and ovarian cancer risk. Results from previous meta-analyses differed. Whereas some studies [4, 10] found positive yet statistically non-significant results, others [8, 9] reported a positive and statistically significant association. We saw the necessity to conduct a comprehensive analysis of all available studies, since the most recently published meta-analysis [8] contained several methodological shortcomings. First, several risk estimates included in that meta-analysis [8] could not be found in the original studies. Second, physical inactivity was used synonymously with sedentary behaviour as exposure variable. This led to the inclusion of one study that solely assessed physical inactivity [34]. We paid particular attention to not equate sedentary behaviour with absence of physical activity. Specifically, we defined physical inactivity as exclusion criterion and excluded four studies from our analysis (Online Resource 3). Third, three publications from the same cohort [16, 39, 40] were included in the aforementioned meta-analysis [8], not only the most recent and updated one [16] but also the two prior publications [39, 40], which may have given that cohort undue weight, potentially distorting the results.

To our knowledge, the current study is the first meta-analysis on the association between sedentary behaviour and ovarian cancer risk that considered the E-Value as a parameter for unmeasured confounding, representing a novel methodological contribution to the existing evidence. The E-Value, a sensitivity analysis for unmeasured confounding, is a relatively new way to test the robustness of an association between exposure and outcome and to evaluate evidence for causation. Even though most of our included studies reduced confounding by adjusting the risk estimates for relevant factors, there is still concern about possible bias caused by uncontrolled confounding. Our results show that an unobserved confounder would have needed to be related to both the exposure and the outcome with a risk ratio of 1.90 to fully explain away the mean RR of 1.29. Also, a confounder would have needed a risk ratio of 1.34 to render the risk estimate statistically non-significant. Although the observed E-value implies a true exposure-outcome association, we cannot fully rule out the existence of an unmeasured confounder associated with sedentary behaviour and ovarian cancer [24].

We were unable to examine whether prolonged sedentary behaviour affects ovarian cancer risk through an etiologic pathway involving obesity because none of the included studies provided risk estimates for different adiposity groups. However, several plausible hypotheses exist regarding obesity as a potential underlying biological mechanism. For example, time spent sedentary replaces time spent with physical activity, and high amounts of sedentary behaviour may coexist with increased energy supply [7], subsequently leading to weight gain and obesity [30, 41]. Current literature indicates that obesity not only increases the risk of ovarian cancer by itself, but may also act as an intermediate variable linking sedentary behaviour to ovarian cancer [42, 43]. Sedentary behaviour and obesity are associated with a rise in sex hormone levels, particularly oestrogen and its metabolites produced by peripheral adipose tissue. This likely facilitates the development and progression of ovarian cancer through mitogenic and mutagenic effects [41, 44, 45]. Adipokines might also affect carcinogenesis through their roles in oestrogen biosynthesis and activity [44, 45]. In addition, sedentary behaviour and obesity-related insulin resistance, higher circulating levels of insulin and glucose, and enhancement of insulin-like growth factor 1 (IGF-1) are likely to boost cancer growth through involvement in cell differentiation, proliferation and apoptosis [41, 46]. Furthermore, systemic chronic inflammation is positively related to sedentary behaviour and obesity [47, 48]. Secretion of proinflammatory adipokines such as tumour necrosis factor-α (TNF-α) and interleukin-6 (IL-6) by dysfunctional adipose tissue [49] and an increase in inflammatory factors, such as C-reactive protein (CRP) [50], are likely to promote cancer development [49]. Future studies of ovarian cancer risk stratified by levels of BMI are needed to formally test whether obesity or its metabolic sequelae represent intermediate steps in the causal pathway linking prolonged sedentary behaviour to ovarian cancer.

One challenge in interpreting the positive association between sedentary behaviour and ovarian cancer observed in our study is that there is currently limited evidence of an inverse relation of physical activity to ovarian cancer [51]. Numerous biologic mechanisms with cancer are believed to operate through an inverse association between physically active and sedentary behaviours. However, prolonged sedentary behaviour is distinct from the absence of physical activity, and time spent sedentary can co-exist with high levels of physical activity [7]. Also, sedentary behaviour may produce physiologic effects independent of those generated by moderate to vigorous activity. For example, several deleterious effects of prolonged sedentary time on cardiometabolic health have been reported for adults who met or exceeded the physical activity recommendations [52]. Thus, an increase in ovarian cancer risk associated with sedentary behaviour may in part be mediated by biologic mechanisms that do not involve an inverse relation of physical activity to ovarian cancer.

Our meta-analysis has numerous important strengths. We used unified and a priori defined criteria for our comprehensive literature search and extraction of relevant information from included studies. We conducted stratified analyses to identify potential sources of heterogeneity and provided precise and valid risk estimates throughout. Notably, we excluded studies that assessed physical inactivity instead of sedentary behaviour as the exposure variable. Importantly, sensitivity analysis for unmeasured cofounding, the E-Value has not yet been conducted in other meta-analyses covering the association between sedentary behaviour and ovarian cancer.

However, there are some limitations to our meta-analysis. Firstly, study designs were heterogenous (prospective cohort and case–control studies). As the number of existing prospective cohort studies on this topic is still small, more high-quality research is required to confirm our results. In addition, almost all studies used self-administered questionnaires [14, 16, 18] or standardised interviews [13, 15, 17] to assess sedentary behaviour instead of objective measures, such as accelerometry. This may have misclassified the true extent of sedentary behaviour [6, 53]. One study [19] used job title assignment to assess sedentary behaviour, which may have resulted in an inaccurate reflection of the actual time spent in sedentary pursuit, due to possible within-job variation, changes in job requirements over time or seasonal changes [54]. These limitations may have led to a certain level of measurement error, but risk estimates would have tended to be underestimated instead of overstated by non-differential misclassification of sedentary behaviour levels [6]. Thus, the true detrimental effect of sedentary behaviour on ovarian cancer risk may be stronger than estimated in our analysis. The analysed data do not allow us to make any assumptions about the association between sedentary behaviour and tumour biology such as specific biological characteristics of ovarian cancer. We found some between-study heterogeneity in the definition of high versus low levels of sedentary behaviour. Addressing this shortcoming by conducting dose–response analyses was not feasible due to the small number of included studies. Caution must be exercised when interpreting our results regarding potential effect measure modification of the sedentary behaviour and ovarian cancer relation because some of our stratified analyses were limited by small sample sizes. Also, insufficient data was the reason we were unable to assess the risk of each histological subtype separately, as only one included study analysed different subtypes of ovarian cancer [16]. Future research should take this into account and consider differentiating between histological subtypes.

In conclusion, this quantitative analysis of all available studies indicates that sedentary behaviour increases the risk of ovarian cancer. Our results represent an important step towards considering sedentary behaviour as a modifiable risk factor for ovarian cancer. Therefore, endeavours to reduce time spent sedentary throughout our everyday lives are to be encouraged on an individual and public health level.

## Supplementary Information

Below is the link to the electronic supplementary material.

Supplementary file 1 (PDF 122 kb)

Supplementary file 2 (PDF 133 kb)

## Data Availability

Not applicable.

## Code availability

Available from the authors on request.
