# Supplementary material for: Sedentary behaviour in relation to ovarian cancer risk: a systematic review and meta-analysis
Source: Eur J Epidemiol. 2021 Jan 25;36(8):769–80. doi: 10.1007/s10654-020-00712-6 (PMC8417015; doi:10.1007/s10654-020-00712-6)
Supplement: Supplementary file 2 — (PDF 133 kb) [file 10654_2020_712_MOESM2_ESM.pdf]

European Journal of Epidemiology

Sedentary behaviour in relation to ovarian cancer risk: a systematic review and meta-analysis

Veronika S. Biller[1], Michael F. Leitzmann[1], Anja M. Sedlmeier[1], Felix F. Berger[1], Olaf Ortmann[2], Carmen Jochem[1]

1) Department of Epidemiology and Preventive Medicine, University of Regensburg, Germany

2) Department of Gynaecology and Obstetrics, University of Regensburg, Germany

Correspondence to:

Ms. Veronika S. Biller

Department of Epidemiology and Preventive Medicine

University of Regensburg

Franz-Josef-Strauss-Allee 11

Regensburg, 93053

Germany, DE

Phone: +49 941 944-5201

E-Mail: veronika.biller@stud.uni-regensburg.de

ORCID-ID: 0000-0003-4506-4895

Online Resource 2 Full search strategy for PubMed

Database last searched on 25 February 2020

Set	Search Terms
Sedentary behaviour and its synonyms	(sedentary behavior OR sedentary behaviour OR sedentary lifestyle) OR (prolonged sitting time OR time spent sitting OR time-spent sitting OR sitting time OR lying time[title/abstract]) OR (leisure time OR leisure activities[MeSH] OR leisure time OR leisure time sitting OR leisure-time sitting OR leisure-time spent sitting OR leisure time spent sitting) OR (computer use[title/abstract] OR computer usage[title/abstract] OR computer time[title/abstract] OR internet use[title/abstract] OR internet usage[title/abstract] OR internet time[title/abstract]) OR (television viewing OR television watching OR television time OR tv viewing OR tv watching OR TV time[title/abstract] OR watch television OR view television OR watch tv OR view tv OR screen watching[title/abstract] OR screen use[title/abstract] OR screen time OR screen entertainment OR screen behaviour OR screen behavior OR screen-based entertainment OR screen-based behaviour OR screen-based behavior) OR (computer game time[title/abstract] OR computer gaming time[title/abstract] OR video game time[title/abstract] OR video gaming time[title/abstract] OR computer game[title/abstract] OR computer gaming[title/abstract] OR video game[title/abstract] OR video gaming[title/abstract]) OR (Automobile driving[MeSH] OR transport time) OR (Reading[MeSH] OR reading time) OR Rest[MeSH]
Ovarian Cancer	AND (Ovarian Neoplasms[Mesh] OR site-specific[title/abstract] OR site specific[title/abstract] OR site-specific cancer incidence[title/abstract] OR site specific cancer incidence[title/abstract])
Limit: Exclude irrelevant publication type	NOT (editorial[ptyp] OR comment[ptyp] OR letter[ptyp] OR guideline[ptyp] OR news[ptyp])
Include Human studies only	AND Humans[MeSH]
Include ovarian cancer incidence as outcome	AND (risk[title/abstract] OR incidence[title/abstract] OR cancer incidence[title/abstract])

Online Resource 3 List of 16 full-text articles assessed for eligibility that were excluded from the analysis with reasons of exclusion

First author, year	Study design	Exclusion criteria
Lee, 2019 [1]	Meta-analysis	Incompatible study design: meta-analysis
Minlikeeva et al., 2019 [2]	Meta-analysis	Incompatible exposure: physical inactivity<br>Incompatible outcome: ovarian cancer survival<br>Incompatible study design: meta-analysis
Lynch et al., 2018 [3]	Meta-analysis	Incompatible study design: meta-analysis
Rangul et al., 2018 [4]	Prospective cohort study	No data on ovarian cancer incidence
Abbott et al., 2016 [5]	Case-control study	Incompatible exposure: physical activity
Cannioto et al., 2016 [6]	Meta-analysis	Incompatible exposure: physical inactivity<br>Incompatible study design: meta-analysis
Cannioto et al., 2016 [7]	Meta-analysis	Incompatible exposure: physical inactivity<br>Incompatible outcome: ovarian cancer mortality<br>Incompatible study design: meta-analysis
Biswas et al., 2015 [8]	Meta-analysis	No data on ovarian cancer incidence<br>Incompatible study design: meta-analysis
Patel et al, 2015 [9]	Prospective cohort study	Updated by a more recent publication
Park et al., 2014 [10]	Prospective cohort study	Incompatible exposure: physical inactivity<br>No data on ovarian cancer incidence
Schmid, Leitzmann, 2014 [11]	Meta-analysis	Incompatible study design: meta-analysis
Shen et al., 2014 [12]	Meta-analysis	Incompatible study design: meta-analysis
Friedenreich et al., 2010 [13]	Systematic review	Incompatible study design: systematic review
Lynch et al., 2010 [14]	Systematic review	Incompatible study design: systematic review
Permuth-Wey et al., 2009 [15]	Systematic review	Incompatible study design: systematic review
Patel et al., 2006 [16]	Prospective cohort study	Updated by a more recent publication

Online Resource 4 Funnel plot for overall sedentary behaviour and risk of ovarian cancer; all analysed studies included, trim-and-fill method applied

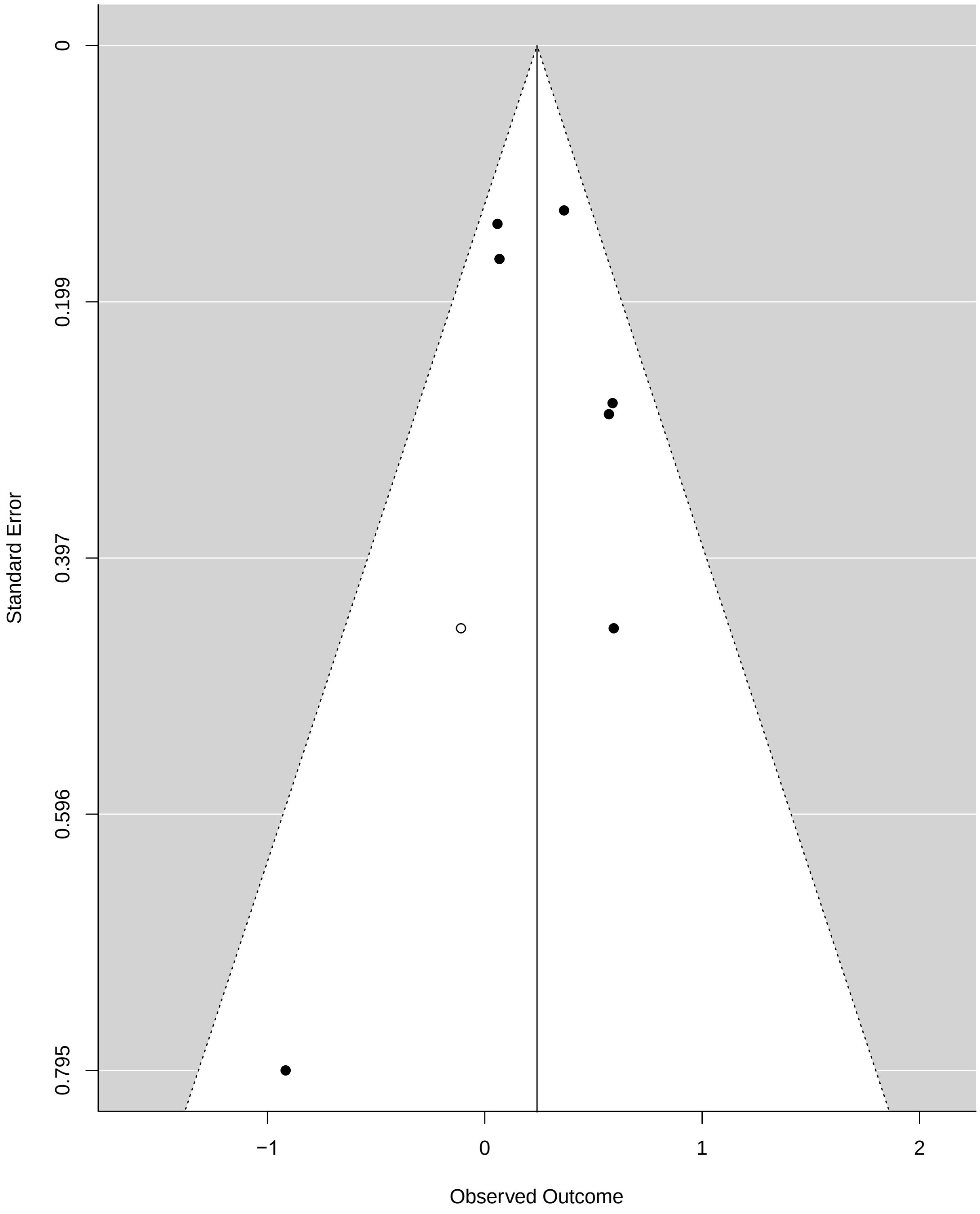

Online Resource 5 Impact of varying degrees of unmeasured confounding bias on the proportion of studies with true risk ratio > 1.1. RR, risk ratio

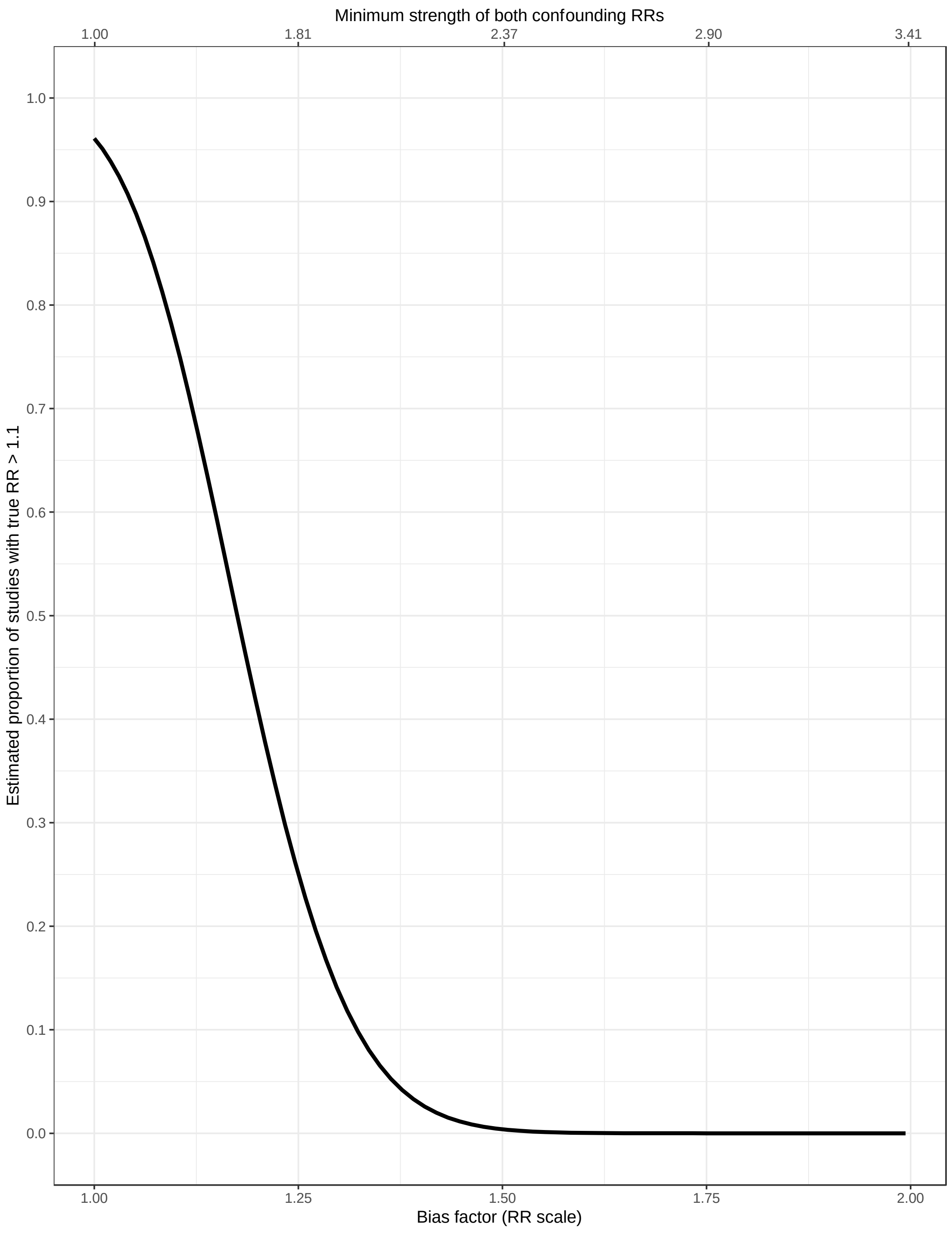

Online Resource References

1. Lee J. Physical activity, sitting time, and the risk of ovarian cancer: A brief research report employing a meta-analysis of existing. Health Care for Women International. 2019;40(4):433-58. doi:10.1080/07399332.2018.1505892.
2. Minlikeeva AN, Cannioto R, Jensen A, Kjaer SK, Jordan SJ, Diergaarde B et al. Joint exposure to smoking, excessive weight, and physical inactivity and survival of ovarian cancer patients, evidence from the Ovarian Cancer Association Consortium. Cancer causes & control : CCC. 2019;30(5):537-47. doi:10.1007/s10552-019-01157-3.
3. Lynch BM, Mahmood S, Boyle T. Sedentary Behaviour and Cancer. In: Leitzmann MF, Jochem C, Schmid D, editors. Sedentary Behaviour Epidemiology. Springer Series on Epidemiology and Public Health. , 2018. p. 245 - 98.
4. Rangul V, Sund ER, Mork PJ, Røe OD, Bauman A. The associations of sitting time and physical activity on total and site-specific cancer incidence: Results from the HUNT study, Norway. PLOS ONE. 2018;13(10):e0206015. doi:10.1371/journal.pone.0206015.
5. Abbott SE, Bandera EV, Qin B, Peres LC, Moorman PG, Barnholtz-Sloan J et al. Recreational physical activity and ovarian cancer risk in African American women. Cancer Med. 2016;5(6):1319-27. doi:10.1002/cam4.677.
6. Cannioto R, LaMonte MJ, Risch HA, Hong C-C, Sucheston-Campbell LE, Eng KH et al. Chronic Recreational Physical Inactivity and Epithelial Ovarian Cancer Risk: Evidence from the Ovarian Cancer Association Consortium. Cancer epidemiology, biomarkers & prevention : a publication of the American Association for Cancer Research, cosponsored by the American Society of Preventive Oncology. 2016;25(7):1114-24. doi:10.1158/1055-9965.EPI-15-1330.
7. Cannioto RA, Lamonte MJ, Kelemen LE, Risch HA, Eng KH, Minlikeeva AN et al. Recreational physical inactivity and mortality in women with invasive epithelial ovarian cancer: evidence from the Ovarian Cancer Association Consortium. British journal of cancer. 2016;115(1):95-101. doi:10.1038/bjc.2016.153.
8. Biswas A, Oh PI, Faulkner GE, Bajaj RR, Silver MA, Mitchell MS et al. Sedentary Time and Its Association With Risk for Disease Incidence, Mortality, and Hospitalization in Adults. Annals of Internal Medicine. 2015;162(2):123. doi:10.7326/m14-1651.
9. Patel AV, Hildebrand JS, Campbell PT, Teras LR, Craft LL, McCullough ML et al. Leisure-Time Spent Sitting and Site-Specific Cancer Incidence in a Large U.S. Cohort. Cancer epidemiology, biomarkers & prevention : a publication of the American Association for Cancer Research, cosponsored by the American Society of Preventive Oncology. 2015;24(9):1350-9. doi:10.1158/1055-9965.EPI-15-0237.
10. Park S, Kim Y, Shin H-R, Lee B, Shin A, Jung K-W et al. Population-attributable causes of cancer in Korea: obesity and physical inactivity. PloS one. 2014;9(4):e90871-e. doi:10.1371/journal.pone.0090871.
11. Schmid D, Leitzmann MF. Television viewing and time spent sedentary in relation to cancer risk: a meta-analysis. J Natl Cancer Inst. 2014;106(7). doi:10.1093/jnci/dju098.
12. Shen D, Mao W, Liu T, Lin Q, Lu X, Wang Q et al. Sedentary behavior and incident cancer: a meta-analysis of prospective studies. PLoS One. 2014;9(8):e105709. doi:10.1371/journal.pone.0105709.
13. Friedenreich CM, Neilson HK, Lynch BM. State of the epidemiological evidence on physical activity and cancer prevention. Eur J Cancer. 2010;46(14):2593-604. doi:10.1016/j.ejca.2010.07.028.
14. Lynch BM. Sedentary behavior and cancer: a systematic review of the literature and proposed biological mechanisms. Cancer epidemiology, biomarkers & prevention : a publication of the American Association for Cancer Research, cosponsored by the American Society of Preventive Oncology. 2010;19(11):2691-709. doi:10.1158/1055-9965.EPI-10-0815.
15. Permuth-Wey J, Sellers TA. Epidemiology of ovarian cancer. Methods Mol Biol. 2009;472:413-37. doi:10.1007/978-1-60327-492-0_20.
16. Patel AV, Rodriguez C, Pavluck AL, Thun MJ, Calle EE. Recreational physical activity and sedentary behavior in relation to ovarian cancer risk in a large cohort of US women. Am J Epidemiol. 2006;163(8):709-16. doi:10.1093/aje/kwj098.
